# Ready for What's Next? The Associations Between Social, Emotional, and Behavioral Skills and Career Adaptability in High School Students

**DOI:** 10.1002/jad.12486

**Published:** 2025-02-28

**Authors:** Gerardo Pellegrino, Tommaso Feraco, Chiara Meneghetti, Barbara Carretti

**Affiliations:** ^1^ Department of General Psychology University of Padova Padova Italy

**Keywords:** 21st century skills, adolescence, career adaptability, career and life development, school‐to‐work transitions, soft skills

## Abstract

**Introduction:**

Transitioning from school to higher education or work is a pivotal moment in a student's life, requiring life‐changing decisions. During this period, students who have acquired a wide range of Social, Emotional, and Behavioral (SEB) skills may feel more confident regarding their capacity to adapt positively to future challenges and difficulties. Our study aimed to test whether five SEB skills domains (self‐management, innovation, social engagement, cooperation, and emotional resilience skills) were associated with four career adaptability resources (i.e., concern, control, curiosity, and confidence).

**Methods:**

A cross‐sectional study was conducted on 350 Italian students (143 boys; *M*_age_ = 16.65) at the end of their last or penultimate year of high school (May–June 2024). We ran a multivariate regression analysis to examine specific associations between SEB skills domains and career adaptability resources. Furthermore, we tested a structural equation model including a latent factor of career‐adaptability as the dependent variable, and SEB skills domains as predictors.

**Results:**

Our results showed that students who described themselves as more competent in self‐management, innovation, and social engagement skills reported higher levels of career adaptability (considering both the latent factor and the specific resources).

**Conclusions:**

Our findings highlight the importance of feeling competent in different domains, not just academics, as the perception of having tools and skills could make students more confident in their ability to face future challenges and adapt to their future careers.

## 1 Introduction

In recent years, the world of work has become less clearly defined and predictable, with an increasing prevalence of precarious employment (Benach et al. 2014; Pförtner 2023), which is characterized by employment insecurity (e.g., fixed‐term contracts, temporary agency work) and income inadequacy (e.g., low wages). Consequently, students need to be prepared to anticipate and navigate transitions and difficulties, as jobs are more temporary and career trajectories are increasingly dynamic (Santilli et al. 2017; Savickas et al. 2009). Furthermore, students must acquire professional competencies that diverge significantly from the abilities and expertise demanded in 20th‐century jobs (Pellegrino and Hilton 2012; Santilli et al. 2017). These skills are referred to in the literature by a variety of names, including soft skills, 21st‐century skills, character strengths, or, more recently, social, emotional, and behavioral (SEB) skills (Feraco et al. 2022; Napolitano et al. 2021). Despite different names and taxonomies, these skills are commonly defined as the nontechnical competencies that every student must develop and train, as they will be required in every career path (Pellegrino and Hilton 2012). Various national and international institutions are trying to understand which skills are most crucial for future work success (European Commission 2016; Ministry of Education, University and Research, MIUR 2018; OECD 2021; World Economic Forum 2016). Their objective is to foster educational practices specifically designed to target these skills and make students more prepared for their future professional endeavors.

Although there is a consensus that SEB skills are crucial for students' professional advancement (Robles 2012), only a few studies (De Guzman and Choi 2013; Oliveira et al. 2023) have yet attempted to correlate these skills with other constructs typically associated with the school‐to‐work or higher education transition. In this study, we focus on the relationship between SEB skills and career adaptability, a crucial aspect of students' preparedness to navigate anticipated and unexpected career transitions and changes (Savickas et al. 2009; Savickas and Porfeli 2012). Career adaptability can be defined as the attitudes and psychological resources that enable individuals to cope with transitions and embrace new educational or career paths (Marciniak et al. 2022). In this study, we hypothesize that students with higher levels of SEB skills might also report higher levels of career adaptability. Possessing diverse skills may foster a sense of capability in navigating the challenges and obstacles associated with the transition to higher education or work. This may, in turn, make students feel more prepared for their future.

### 1.1 Social, Emotional, and Behavioral Skills: A New Theoretical Framework

Research on SEB skills has always been constrained by a proliferation of theoretical models and a lack of reliable measures to assess them (Abrahams et al. 2019). To overcome these limitations, Soto and colleagues (2022a) have developed a novel framework for SEB skills and operationalized a new measure to assess them, the Behavioral, Emotional, and Social Skills Inventory—BESSI.

According to this model, SEB skills are defined as individuals' capacities to build and maintain social relationships, regulate emotions, manage goal‐directed behaviors, and learn from new experiences (Napolitano et al. 2021; Soto et al. 2022a). After reviewing several SEB skills taxonomies, the authors noticed that most of the skills identified in literature could be linked to the Big Five personality traits (McCrae and Costa 1989). In particular, five major skill domains have been identified:

‐ Self‐management skills (e.g., goal regulation, decision‐making skill, time management): related to the Big Five domain of conscientiousness, they represent the capacities used to effectively set and achieve goals.
‐ Innovation skills (e.g., creative skill, cultural competence): related to the Big Five domain of openness, they represent the capacities used to engage with novel experiences and ideas.
‐ Social engagement skills (e.g., conversational skill, leadership): related to the Big Five domain of extraversion, they represent the capacities to actively engage with other people.
‐ Cooperation skills (e.g., teamwork skill, perspective‐taking skill): related to the Big Five domain of agreeableness, they represent the capacities used to build and maintain positive social relationships.
‐ Emotional resilience skills (e.g., stress regulation, capacity for optimism): related to the Big Five domain of neuroticism, they represent the capacities used to regulate emotions.

Despite the similarities, SEB skills are distinct from personality traits: while the latter represent how a person tends to feel, think, and behave in different situations, skills represent how a person can think, feel, and behave when they want or need to do so (Soto et al. 2022a). For example, an introverted student may prefer to study and work alone. However, if this student is assigned to a group task, they may still possess the conversational and expressive skills to engage positively with other team members. On the other hand, an extroverted student, who generally tends to participate actively in group activities, may not always use these skills effectively in a group context (e.g., they may struggle to communicate their thoughts and feelings clearly).

The distinction between skills and personality traits is relevant. Skills are generally more malleable than traits and can undergo changes throughout life experiences (Napolitano et al. 2021). Conversely, traits demonstrate greater stability and are less susceptible to modification. For this reason, more emphasis should be placed on developing skills in the educational context, as this can have a significant impact on student outcomes (Feraco et al. 2022).

Indeed, SEB skills have already proved to be associated with important educational and individual outcomes such as student engagement, academic achievement, well‐being, life satisfaction, relationships with peers, and occupational interests (Sewell et al. 2023; Soto et al. 2022a, 2022b; Yoon et al. 2024). However, to date, only one study has considered work‐related outcomes. Chen et al. (2024) observed a positive association between SEB skills and both organizational citizenship behaviors and job satisfaction. These findings suggest that further investigation into the role of SEB skills beyond educational outcomes is warranted.

A further step should be understanding whether these skills support students during important transition moments, such as the end of high school. Upon completing their secondary education, students may consider the competencies they have developed, both academic and nonacademic, and contemplate how they might apply these abilities in future professional endeavors. In particular, students who have developed high levels of SEB skills in different domains may feel more confident in facing the obstacles associated with the transition from school to work or higher education and feel more prepared for that moment. For this reason, SEB skills might be associated with other important psychological resources that support students when facing moments of transition, such as career adaptability resources (Savickas et al. 2009; Savickas and Porfeli 2012).

### 1.2 Career Adaptability at the End of High School

The end of high school marks a fundamental moment of transition, as students have to make important decisions concerning their future careers. Such experience can result in feelings of anxiety and uncertainty, which may impede the student's ability to make well‐informed decisions (Vignoli 2015). To successfully navigate the stress and challenges associated with this transition, students must draw upon a range of internal psychological resources. Among these, career adaptability is undoubtedly a crucial factor that has been studied concerning both school‐work and university‐work transitions (Marciniak et al. 2022; Merino‐Tejedor et al. 2016; Steiner et al. 2019). According to the career construction theory (Marciniak et al. 2022; Savickas 2005; Savickas et al. 2009), career adaptability is a key component of career preparedness and denotes an individual's psychological resources and attitudes necessary for coping with current and anticipated vocational development tasks, transitions, and work traumas that alter their social integration to some degree. Students with higher levels of career adaptability usually have better outcomes in terms of academic performance and satisfaction, life satisfaction, and various career‐related outcomes (e.g., career identity, job satisfaction, calling, and organizational commitment; Marciniak et al. 2022; Negru‐Subtirica and Pop 2016; Rudolph et al. 2017).

Adaptability resources include the self‐regulation strengths or capacities that individuals use to cope with career‐related changes and to solve complex problems that arise during their occupational transition, and include four global dimensions: concern, control, curiosity, and confidence (Savickas et al. 2009; Savickas and Porfeli 2012). Career *concern* means a future orientation, a sense that it is important to prepare for tomorrow. Concerned individuals are aware of the vocational tasks and occupational transitions to be faced and choices to be made in the imminent and distant future. Career *control* involves intrapersonal self‐discipline and the process of being deliberate, organized, independent, and decisive in making occupational transitions. Control disposes students to responsibly engage and negotiate transitions, rather than avoid them. Career *curiosity* refers to the initiative to learn about the types of careers that one might want to do and the exploration of the fit between oneself and the work world. Curious students are open to new experiences and ready to experiment with various situations and roles. Finally, career *confidence* denotes the anticipation of success in encountering challenges and overcoming obstacles. To summarize, students with higher adaptability care more about their future, feel in control of their occupational choices, are curious about their possible occupational role, and are optimistic about their abilities to face difficulties in moments of transition (Marciniak et al. 2022; Santilli et al. 2017; Savickas et al. 2009).

The construct of career adaptability has been extensively studied, and much focus has been given to individual (e.g., personality traits, self‐esteem, academic self‐efficacy) and contextual (e.g., family background, social support, networking opportunities) factors associated with these psychological resources (Marciniak et al. 2022; Rudolph et al. 2017). However, only a few studies have explored the possible relations between career adaptability and SEB skills. In a correlational study on high school students, Oliveira et al. (2023) explored the associations between nine social‐emotional skills (i.e., tolerance, trust, empathy, responsibility, optimism, curiosity, assertiveness, cooperation, and sociability) drawn from the OECD (2021) framework and career adaptability. All the skills considered, except responsibility, were positively associated with career adaptability. However, the authors reported only the bivariate correlations between the variables, which limits the ability to understand the unique contribution of each variable when accounting for the influence of the others. Without considering multivariate analyses, it is difficult to determine the relative importance of each factor in the context of the others. In another study (De Guzman and Choi 2013), three employability skills (i.e., communication, problem‐solving, and teamwork skills), which partly overlap with SEB skills, were positively related to career adaptability and its dimensions. Despite these promising findings, further investigation is necessary to elucidate the relationship between career adaptability and SEB skills. These studies, in fact, only considered specific skills, without adopting a comprehensive framework, and this may pose a limitation to the generalizability of the results (Abrahams et al. 2019). Conversely, a comprehensive framework acknowledges the organization of skills within broader domains, reflecting their structure and interrelations (Soto et al. 2022a). This approach enables the detection of the specific effects of each skill domain while accounting for the influence of other domains. Additionally, the adoption of a common and comprehensive framework facilitates the accumulation and integration of knowledge and standardizes the way skills are measured and defined.

The recently proposed SEB skills framework by Soto and colleagues (2022a) may offer a solution, as it has combined in a psychometrically robust and theoretically founded framework, the main skills that have been considered in previous literature. Moreover, the SEB skills framework and the inventory to measure them (the BESSI) have been translated and validated in different cultural contexts (Feraco et al. 2024; Lechner et al. 2022; Postigo et al. 2024). For these reasons, this framework may prove valuable in developing a more detailed understanding of the role that SEB skills play in supporting the transition from school to work or from school to university.

### 1.3 Rationale of the Study and Hypotheses

The present study posits that students who perceive that they have developed a diverse range of SEB skills across different domains also feel more prepared to face the challenges associated with the transition to higher education or the workforce. Possessing such skills may contribute to a sense of preparedness and confidence at the conclusion of their academic journey, as they are equipped with a toolbox of competencies that they can use to navigate future obstacles and difficulties (Napolitano et al. 2021).

To accomplish this aim we planned a cross‐sectional design examining relations between SEB skills and career adaptability factors in students in their second‐to‐last or last year of high school. These students are confronted with a multitude of decisions regarding their future trajectory, including the choice between pursuing higher education and/or entering the workforce. Although this study employs a cross‐sectional design, it represents a critical first step in understanding the associations between SEB skills and career adaptability among adolescents and in guiding future longitudinal research.

We expected positive associations between the five SEB skills domains and career adaptability resources. In particular, we expected the following associations:

‐ All five SEB skills (self‐management, innovation, social engagement, cooperation, and emotional resilience) are associated with career confidence: SEB skills might represent the building blocks of a more general sense of self‐efficacy (Feraco et al. 2024), which could enhance students' confidence in navigating transitions and associated challenges.
‐ Self‐management skills are significantly associated with the dimensions of career concern, control, and confidence. Self‐management skills are indeed core competencies that allow students to make plans for their future, set goals, be persistent in achieving them, and responsibly make informed decisions (Napolitano et al. 2021; Soto et al. 2022a).
‐ Innovation skills are associated with career concern and curiosity. Students with higher levels of innovation skills might be more capable of engaging with novel situations and experiences (Napolitano et al. 2021; Soto et al. 2022b). Consequently, they might be more oriented toward their occupational future (concern) and more interested in exploring possible roles and career alternatives (curiosity).
‐ Social engagement and cooperation skills are associated with career concern and curiosity. These skills may facilitate positive relations with peers, parents, and teachers, thereby allowing the student to establish a robust network of social support. This, in turn, may assist students in addressing their concerns, discussing future plans, and sharing diverse ideas and possibilities (Marciniak et al. 2022).

Given the limited existing literature on the topic (De Guzman and Choi 2013; Oliveira et al. 2023), and the lack of adoption of the SEB skills framework in this context (Napolitano et al. 2021), our hypotheses were primarily theoretically based. Consequently, while we prioritized certain associations based on theoretical considerations (Marciniak et al. 2022; Napolitano et al. 2021; Soto et al. 2022a, 2022b; Savickas and Porfeli 2012), we treated other possible associations as exploratory and included them in our analysis design.

Given that career adaptability is often considered a global factor encompassing all four resources (Savickas and Porfeli 2012), we further tested the associations between SEB skills and a latent score of career adaptability. We expected a positive significant association between self‐management, innovation, social engagement, and cooperation skills, given that they might play a role with more than one adaptability resource.

Academic achievement (school grades) and self‐efficacy were added as covariates, given their positive association with career adaptability (Marciniak et al. 2022; Yon et al. 2012; Zeng et al. 2022). We hypothesized that SEB skills were associated with career adaptability resources even after accounting for academic achievement and self‐efficacy. For high school students, it is not only important to perceive oneself as competent in academic domains; it is also crucial to perceive the acquisition of a wide range of skills that may prove useful beyond the academic domain.

## 2 Materials and Methods

### 2.1 Participants

#### 2.1.1 Power Analysis

To ensure sample size adequacy, we conducted a power analysis via simulation, running 10,000 iterations per sample size. We estimated medium regression coefficients (*β* = 0.20) based on our literature review (Marciniak et al. 2022; Rudolph et al. 2017; Soresi et al. 2012; Soto et al. 2022b) and research hypothesis. The power was then calculated specifically for the five regression coefficients representing the effects of the SEB skill domains on the latent factor of career adaptability. Our analysis indicated that a sample size of 290 participants would yield a power of 0.82 for all five effects. A power analysis on specific career adaptability resources was not conducted due to the limited existing literature on the topic, which would have resulted in an uninformative analysis.

#### 2.1.2 Total Sample

A total of 356 students in their last or second‐to‐last year of high school (from 16 to 19 years old) from two Italian schools voluntarily participated in the study. Because gender was added as an independent variable, six participants were excluded from the analysis because they reported a gender other than boy or girl. The final sample consisted of 350 students (143 boys) with a mean age of 16.65 years (SD = 0.67). One hundred and seventy‐six students were enrolled in the fourth year of high school, and 174 in the fifth year. In the Italian educational system, the fourth and fifth years of high school represent the last 2 years of secondary education. After them, students can pursue higher education, vocational schools, or enter the world of work.

### 2.2 Materials

Participants completed an introductory demographic questionnaire containing questions about age, gender, grade, and type of school. The reliability of the questionnaires in our sample was assessed with Cronbach's *α* and McDonald's *ω* using the *semTools* package (Jorgensen et al. 2022; see Table 1).

**Table 1:** Descriptive statistics of all study variables.

		*M*	SD	Skewness	Kurtosis	*α*	*ω*
Age (years)		16.65	0.67	0.30	2.49	—	—
SEB skills	Self−management	3.45	0.57	−0.09	3.76	0.86	0.83
	Innovation	3.29	0.56	0.26	3.86	0.83	0.80
	Cooperation	3.54	0.57	−0.07	3.87	0.84	0.82
	Social engagement	3.21	0.64	0.07	3.38	0.86	0.88
	Emotional resilience	3.02	0.69	0.18	3.28	0.89	0.89
Career adaptability	Concern	3.53	0.75	−0.01	2.99	0.92	0.90
	Control	3.64	0.64	0.16	2.35	0.83	0.81
	Curiosity	3.67	0.63	0.11	2.37	0.89	0.86
	Confidence	3.60	0.62	0.40	2.43	0.90	0.87
Academic outcomes	Achievement	7.33	1.08	−0.12	2.84	—	—
	Academic self−efficacy	3.76	0.67	−0.42	3.09	0.90	0.85

*Note: α* = Cronbach's alpha; *ω* = McDonald's omega.

#### 2.2.1 Social, Emotional, and Behavioral Skills

The Behavioral, Emotional, and Social Skills Inventory—45 (Feraco et al. 2024; Sewell et al. 2024) measures participants' social, emotional, and behavioral skills with 45 items on a 5‐point Likert scale (from 1 = *not at all well* to 5 = *exceptionally well*). Participants report their ability to perform the behavior, thought, or feeling described in each item (e.g., “Plan out my time”, “Lead a group of people”). The BESSI‐45 includes 9 items for each SEB domain (i.e., management, social engagement, cooperation, emotional resilience, and innovation skills). Average scores for each skills domain were calculated separately.

#### 2.2.2 Career Adaptability

The Career Adapt‐Abilities Scale (CAAS; Savickas and Porfeli 2012; Soresi et al. 2012) measures four dimensions of career adaptability—concern, control, curiosity, and confidence—with 24 items on a 5‐point Likert scale (from 1 = *not strong* to 5 = *strongest*). Participants report how strongly they have developed the abilities described in each item (e.g., “Looking for opportunities to grow as a person”, “Making decisions by myself”). The CAAS included six items for each career adaptability dimension. Average scores for each dimension were calculated separately.

#### 2.2.3 Academic Self‐Efficacy

The Academic Self‐Efficacy Questionnaire (De Beni et al. 2014) measures the belief that one can succeed in studying with five items on a 5‐point Likert scale (from 1 = *poor* to 5 = *excellent*). It included five items (e.g., “How do you rate your study skills?”). A single score was computed by averaging the five items.

#### 2.2.4 Academic Achievement

In the Italian school system, students receive grades on a 10‐point scale where six represents a passing grade. Students self‐reported the grades in five core subjects included in all Italian school curricula: Italian, Mathematics, English, Science, and History. Correlations between grades range between *r* = 0.38 and *r* = 0.61. For the analysis, we averaged all grades into a single score.

### 2.3 Procedure

The study was approved by the Ethical Committee of the University of Padova. The researchers contacted the school principals via email, provided detailed explanations of the study's aims and procedures, and invited them to participate in the study. Consent forms were distributed by the schools to the parents, or the students over the age of 18, who signed them before data collection.

All data were collected online using Qualtrics during regular class time. Demographic information was collected at the beginning of the questionnaire, while the questionnaires were presented in randomized order across participants. All instruments required approximately 20 min to complete.

Instructors provided students with the Qualtrics link, read the instructions aloud, and then guided the students through the process. Data were collected in May–June 2024 (in the last weeks of the Italian academic year). This study was part of a larger three‐wave longitudinal study (previous data collections occurred at the beginning of the academic year and the end of the first term) including other measures administered and not utilized in the present study.

### 2.4 Data Analysis

Data, code, and materials are available on OSF at https://osf.io/8thsg/.

All analysis was run using R (R Core Team 2024). Table 1 shows the means, standard deviations, skewness, and kurtosis for all the variables, which were standardized for subsequent analysis. Preliminary correlational analysis was run for all observed variables.

We ran multivariate regression models to examine the associations between the five SEB skills domains and the four career adaptability resources. Grade, gender, academic achievement, and self‐efficacy were added as covariates. We calculated the *R* ^2^ to estimate the variability of the dependent variables explained by the model.

Finally, we ran a structural equation model that was fitted using Maximum Likelihood Estimation (MLE). A latent factor of career adaptability was calculated using the average scores of the four dimensions (concern, control, curiosity, and confidence) and regressed on SEB skills domains. Gender, grade, academic achievement, and self‐efficacy were added as covariates. Model fit was evaluated with four different fit indices: The Comparative Fit Index (CFI), the Tucker Lewis Index (TLI), the Standardized Root Mean Square Residual (SRMR), and the Root Mean Square Error of Approximation (RMSEA). We judged model fit according to the following criteria (Hu and Bentler 1999): CFI and TLI > 0.90 (“adequate”) or > 0.95 (“good”); SRMR and RMSEA < 0.06 (“good”) or < 0.10 (“adequate”).

## 3 Results

Descriptive statistics are reported in Table 1. Correlations among all observed variables included in the study are reported in Table 2. All SEB skills were positively associated with career adaptability resources, with correlational coefficients (Pearson's *r*) ranging between 0.28 and 0.59 (*p* < 0.001).

**Table 2:** Correlations (Pearson's *r*) between all observed variables considered in the study.

		1.	2.	3.	4.	5.	6.	7.	8.	9.	10.
1.	Self‐management										
2.	Innovation	0.54**									
3.	Social engagement	0.55**	0.52**								
4.	Cooperation	0.61**	0.51**	0.63**							
5.	Emotional resilience	0.50 **	0.47**	0.43**	0.43**						
6.	Concern	0.54**	0.46**	0.43**	0.40 **	0.28**					
7.	Control	0.54**	0.45**	0.55**	0.41**	0.36**	0.64**				
8.	Curiosity	0.49**	0.50 **	0.40 **	0.43**	0.28**	0.64**	0.73**			
9.	Confidence	0.59**	0.51**	0.47**	0.44**	0.38**	0.65**	0.72**	0.73**		
10.	Academic achievement	0.37**	0.32**	0.23**	0.24**	0.14*	0.21**	0.24**	0.24**	0.29**	
11.	Academic self‐efficacy	0.59**	0.45**	0.41**	0.39**	0.30 **	0.38**	0.42**	0.40 **	0.49**	0.69**

* *p* < 0.01

** *p* < 0.001.

### 3.1 Multivariate Regression Analysis

**Figure 1:**
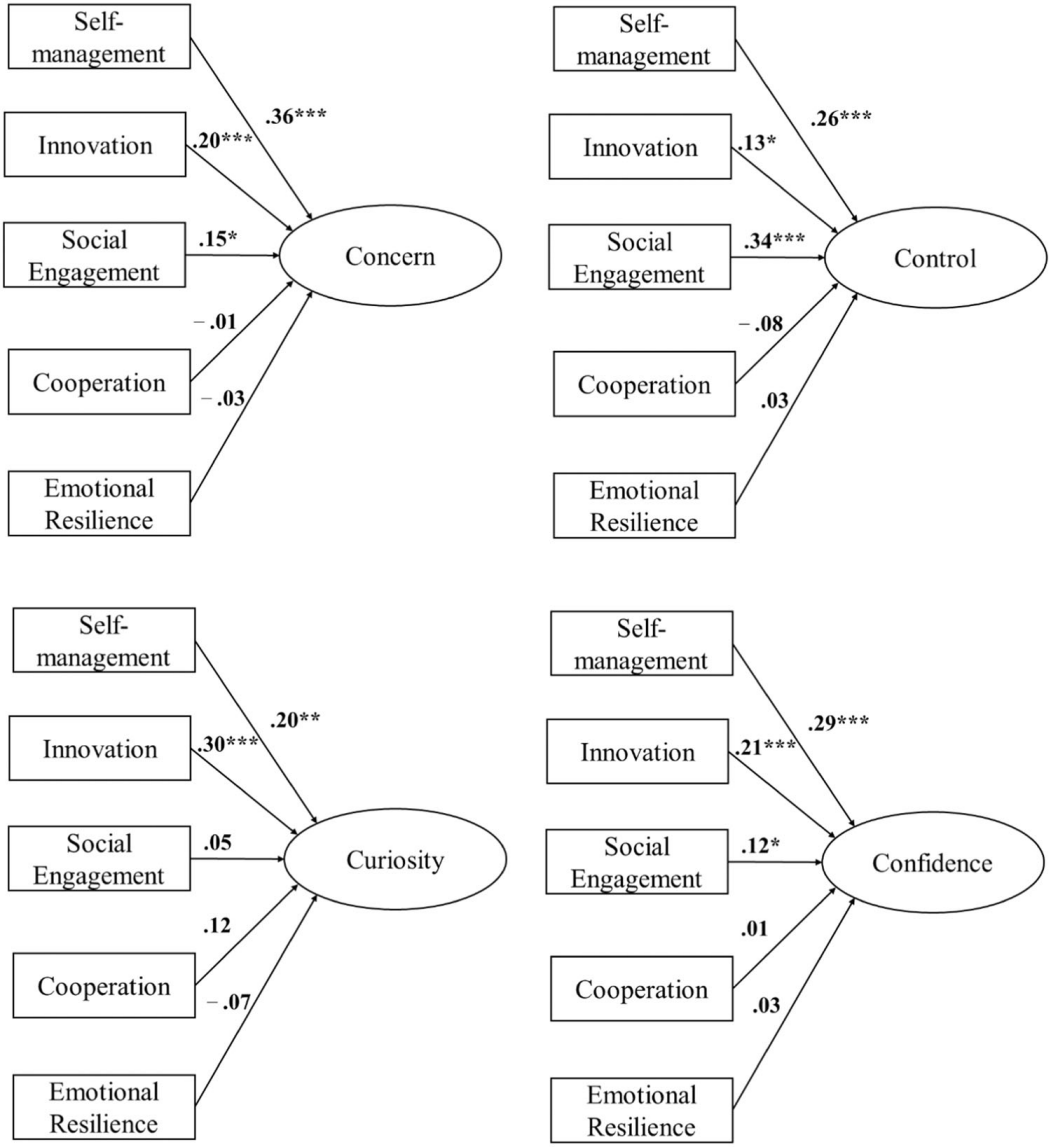
Results of the multivariate regression analysis. Associations between SEB skills and career adaptability dimensions, after controlling for gender, grade, academic achievement, and academic self‐efficacy. *p < 0.05, **p < 0.01, ***p < 0.001.

The associations between SEB skills and career adaptability dimensions are represented in Figure 1. Complete results are reported in Table 3. The model explained 34% of the variance for concern, 39% for control, 32% for curiosity, and 42% for confidence.

**Table 3:** Results of the multivariate regression analysis. Standardized coefficients (*β*) of all predictors on dimensions of career adaptability are reported.

	Dimensions of career adaptability			
	Concern	Control	Curiosity	Confidence
(Intercept)	−0.13	0.03	0.01	0.06
Grade	0.03	−0.13	−0.03	−0.08
Gender	0.20*	0.05	0.01	−0.04
Self‐management	0.36***	0.26***	0.20**	0.29***
Innovation	0.20***	0.13*	0.30***	0.21***
Social engagement	0.15*	0.34***	0.05	0.12*
Cooperation	−0.01	−0.08	0.12	0.01
Emotional resilience	−0.03	0.03	−0.07	0.03
Academic achievement	−0.06	−0.05	−0.06	−0.06
Academic self‐efficacy	0.06	0.12	0.14	0.20**

*Note:* Grade = from 4th and 5th grade; Gender = from boys to girls.

* *p* < 0.05

** *p* < 0.01

*** *p* < 0.001.

Self‐management skills showed a significant positive association with concern (*β* = 0.36, *p* < 0.001), control (*β* = 0.26, *p* < 0.001), curiosity (*β* = 0.20, *p* < 0.01), and confidence (*β* = 0.29, *p* < 0.01).

Innovation skills were significantly associated with concern (*β* = 0.20, *p* < 0.001), control (*β* = 0.13, *p* < 0.05), curiosity (*β* = 0.30, *p* < 0.001), and confidence (*β* = 0.21, *p* < 0.001).

Social engagement skills were significantly associated with concern (*β* = 0.15, *p* < 0.05), control (*β* = 0.34, *p* < 0.001), and confidence (*β* = 0.12, *p* < 0.05).

Academic self‐efficacy showed a significant association with confidence (*β* = 0.20, *p* < 0.01). Girls reported higher levels of career concern (*β* = 0.20, *p* < 0.05).

### 3.2 Structural Equation Model

**Figure 2:**
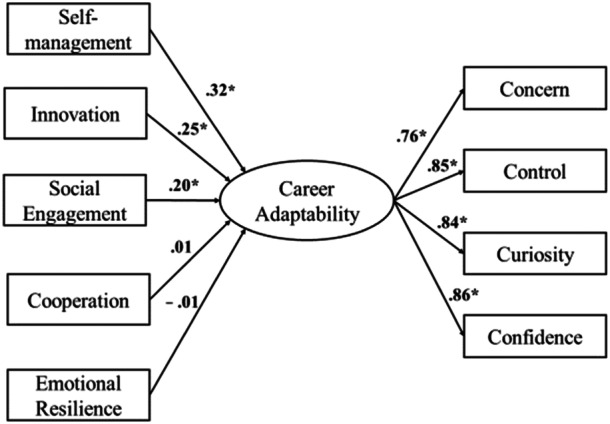
Associations between SEB skills and career adaptability (latent factor), after controlling for gender, grade, academic achievement, and academic self‐efficacy. *p < 0.001.

The associations between SEB skills and career adaptability are represented in Figure 2. Complete results are reported in Table 4. The model displayed adequate fit indices: CFI = 0.95; TLI = 0.93; SRMR = 0.02; RMSEA = 0.07. The four adaptability resources adequately converged into the latent factor of career adaptability, with factor loadings ranging between 0.76 and 0.86 (*p* < 0.001). Self‐management (*β* = 0.32, *p* < 0.001), innovation (*β* = 0.25, *p* < 0.001), and social engagement skills (*β* = 0.20, *p* < 0.001), and academic self‐efficacy (*β* = 0.17, *p* < 0.05) were significantly associated with career adaptability. Overall, the model explained 53% of the variance for career adaptability.

**Table 4:** Results of the structural equation model: factor loadings for the latent variable of career adaptability and regression coefficients of all variables on career adaptability.

			*β*	SE	*z*	*p*	Lower 95% CI	Upper 95% CI
Factor loadings								
Adaptability	→	Concern	0.76	0.02	30.86	< 0.001	0.71	0.81
	→	Control	0.85	0.02	47.30	< 0.001	0.81	0.89
	→	Curiosity	0.84	0.02	45.03	< 0.001	0.80	0.88
	→	Confidence	0.86	0.02	50.47	< 0.001	0.83	0.90
Regression coefficients								
Adaptability	~	Grade	−0.04	0.04	−0.91	0.36	−0.12	0.04
	~	Gender	0.02	0.04	0.49	0.62	−0.07	0.11
	~	Self‐management	0.32	0.06	5.36	< 0.001	0.20	0.44
	~	Innovation	0.25	0.05	4.76	< 0.001	0.15	0.35
	~	Social engagement	0.20	0.06	3.54	< 0.001	0.09	0.31
	~	Cooperation	0.01	0.06	0.17	0.86	−0.10	0.12
	~	Emotional resilience	−0.01	0.05	−0.16	0.88	−0.11	0.10
	~	Academic achievement	−0.07	0.06	−1.17	0.24	−0.18	0.04
	~	Academic self‐efficacy	0.17	0.07	2.53	< 0.05	0.04	0.30

Abbreviations: *β* = standardized coefficient; CI = confidence interval; Gender = from boys to girls; Grade = from 4th to 5th grade; SE = standard error.

## 4 Discussion and Conclusions

Despite the existence of disparate theoretical frameworks, there is a consensus that SEB skills are indispensable for the transition from school to higher education or the world of work (Pellegrino and Hilton 2012; Robles 2012). Nevertheless, only two studies (De Guzman and Choi 2013; Oliveira et al. 2023) have investigated the potential role of SEB skills as individual resources that can support students at the conclusion of their secondary education. However, these studies only focused on specific skills, without considering a more comprehensive theoretical framework.

The objective of our study was to address this gap by establishing a connection between the recent SEB skills framework (Soto et al. 2022a) and another crucial construct in the context of school‐to‐work or higher education transition, namely career adaptability (Savickas and Porfeli 2012). As research on this topic has been scarce, the present correlational study aims to provide preliminary insights into the associations between SEB skills and career adaptability, thereby laying the foundation for future research in this domain.

While career adaptability resources have been described as individual attitudes toward future experiences and transitions (Marciniak et al. 2022; Savickas et al. 2009), SEB skills are behavioral competencies that represent what an individual is capable of doing if a future situation requires it (Napolitano et al. 2021; Soto et al. 2022a). Students who possess strong competencies in various domains may feel more confident about their future career success. Such confidence is derived from the perception of being adequately prepared to handle tasks and challenges, even those that have not been previously encountered. Adolescents with higher levels of SEB skills might adopt a more proactive stance—anticipating problems, setting goals, and engaging in multicultural contexts—thus being better prepared to face challenges and achieve success in their academic, career, and personal lives (Oliveira et al. 2023).

Our findings partially supported our hypothesis. At a correlational level, all five SEB skills domains were positively associated with the four career adaptability resources. However, the multivariate regression models showed that self‐management, innovation, and social engagement skills domains maintained significant associations with career adaptability resources. This result was further confirmed by the SEM model, where these three skills significantly predict the career adaptability latent factor.

More notably, these three domains were found to be positively associated with career adaptability resources even when controlling for academic achievement and self‐efficacy. This indicates that for students approaching the end of their high school education, their expectations regarding their future careers are not solely influenced by their academic performance and self‐perception as successful students. Instead, they are also shaped by their competence in various domains and areas of life, which extend beyond the academic domain.

Among the SEB skills domains, self‐management skills are associated with all those processes of self‐regulation of behavior that are crucial in times of transition (Merino‐Tejedor et al. 2016; Napolitano et al. 2021). For example, these skills help students at the end of high school set long‐term goals (e.g., career goals), create action plans to achieve them, and explore possible alternatives. In general, self‐management skills facilitate students' perception of greater control over forthcoming changes and significant decisions. Consequently, students with higher self‐management skills may also report greater career adaptability resources.

Innovation skills include the ability to manage complex information, think creatively, and learn from new people and experiences (Napolitano et al. 2021). Our findings revealed that these skills are associated with all four resources of career adaptability, and in particular with curiosity: Students who believe they are more capable of learning from new experiences may also be more willing to explore different possible career paths with the expectation that they can derive the best from each experience (Zacher 2014). For these students, transitions are important opportunities to grow and learn new things, making them feel more confident about their future.

Finally, social engagement skills are positively associated with career adaptability, particularly with concern and control. Previous research has highlighted the importance of social support for career adaptability (Marciniak et al. 2022; Perry et al. 2010). Students with higher levels of social engagement skills may have developed robust social networks (Soto et al. 2022b) that allow them to access valuable resources such as practical information, mentoring, and emotional support. These networks may reinforce career adaptability resources by providing students with a clearer understanding of their career interests and abilities, as well as strategies for overcoming challenges (Perry et al. 2010). As a result, students with stronger social engagement skills may not only use social support more effectively but may also use it to strengthen their career adaptability resources.

The results of our study did not support the hypothesis that there is a relationship between cooperation or emotional resilience skills and career confidence. While the bivariate correlations indicated a significant positive association between these two skill domains and all four career adaptability resources, this was not replicated in the multivariate regression and SEM analysis. These skills may become more relevant in later stages, such as at the beginning of a new educational or career path, during which students need to develop and maintain positive relationships with their new colleagues or co‐workers and deal with stressful situations while adapting to their new roles and identities. These hypothesized associations cannot be captured by a cross‐sectional design, an issue that could be addressed in future longitudinal studies.

In conclusion, our findings support the importance of SEB skills for career adaptability, as already shown by previous studies (De Guzman and Choi 2013; Oliveira et al. 2023). By adopting the new theoretical framework of SEB skills (Soto et al. 2022a), our study allows us to better differentiate the role of different skills domains in relation to both a global factor of career adaptability and its four specific dimensions.

## 5 Limitations

It is important to acknowledge the limitations of the present study. First, given the correlational design of the study, it is not possible to understand the causal mechanism of the associations between SEB skills and career adaptability. Furthermore, our study is the first to examine the associations between these variables. Therefore, further research is needed to validate and extend these findings. A longitudinal design could be used to understand how SEB skills and career adaptability resources develop during high school years, providing deeper insights into their relationships. In addition, the results are limited to a specific group of students: Students in their last 2 years of high school. Thus, future research should extend our findings by understanding how SEB skills and career adaptability resources are related to higher education and career outcomes beyond high school. Moreover, the present study included participants who identified as either boy or girl. Even if gender is only partially relevant in the variables considered, future research could consider the full range of gender identities. Finally, only self‐report measures were used to assess SEB skills and career adaptability resources. Although this is the most common approach to measuring these constructs (Abrahams et al. 2019; Savickas and Porfeli 2012), alternative methods, such as incorporating multi‐informant reports (e.g., from teachers and parents), could enhance the validity of the results.

## 6 Implications

The present study has significant theoretical implications, as it integrates two robust theoretical frameworks, the SEB skills framework (Napolitano et al. 2021; Soto et al. 2022a) and career construction theory (Savickas et al. 2009; Savickas and Porfeli 2012), and lays the foundation for further experimental and longitudinal research on this topic. As previous research has already explored the role of several skills in association with career adaptability resources (De Guzman and Choi 2013; Oliveira et al. 2023), our study has the strength of adopting the SEB skills framework, which allows for a clear differentiation of the contribution of the five SEB skills domains in association with career adaptability.

Our study has important practical implications. Helping students in reflecting about their skills is already part of career intervention on students (e.g., Chiesa et al. 2016), but focusing on the specific skills domains that are associated with career adaptability might increase the efficacy of these interventions. Skills are more malleable than personality traits and therefore develop through different experiences, especially during adolescence (Napolitano et al. 2021). It is therefore important for practitioners to acknowledge that, in addition to targeted interventions, SEB skills may be improved during different life experiences (e.g., sports, volunteering, and activities with family and peers; Feraco et al. 2022). For example, being part of a sport team might help the student in developing communication, teamwork, and leadership skills, that could be requested and appreciated in their future career. Reflecting on the competencies developed in informal context might be an additional part of career intervention that might help the students in considering their strengths and abilities besides the academic context. Fostering skills might be the key to making the students more confident about their future and ready to face changes and transitions.

## Ethics Statement

APA ethical standards were followed in the conduct of the study, and approval was granted by the University of Padova Ethical Committee.

## Conflicts of Interest

The authors declare no conflicts of interest.

## Data Availability

Data, code, and materials are available on OSF at https://osf.io/8thsg/.
